# The Spectrum of Testicular Pathologies Upon Scrotal Exploration for Acute Scrotum: A Retrospective Analysis

**DOI:** 10.7759/cureus.10984

**Published:** 2020-10-16

**Authors:** Muhammad Khalid Syed, Ahmad A Al Faqeeh, Alsayed Othman, Ahmed A Hussein, Salman Hussain, Talal Almas, Reema Alsufyani, Hasan Alaeddin, Saifullah Syed, Sabahat K Syed

**Affiliations:** 1 Pediatric Surgery, King Fahad Hospital, Al Baha, SAU; 2 Pediatric Surgery, Al-Azhar University - Assuit Branch, Assuit, EGY; 3 Internal Medicine, Royal College of Surgeons in Ireland, Dublin, IRL; 4 Internal Medicine, Jinnah Sindh Medical University, Karachi, PAK

**Keywords:** scrotal exploration, testicular torsion, testicular anomalies

## Abstract

Background

The term acute scrotum encompasses a plethora of testicular pathologies that are detrimental to the survival and sustenance of testes. The aim of the present study is to determine the spectrum of these testicular pathologies upon scrotal exploration performed in the aftermath of acute scrotal pain in the pediatric population.

Methods

This multicenter retrospective cross-sectional study was conducted at the department of pediatric surgery at two hospitals. During the study period, the clinical characteristics of the 76 patients that underwent scrotal exploration for acute scrotum were assessed. These included age, duration of symptoms on presentation, and identification of the etiology underlying scrotal pain. The data obtained was eventually analyzed using the Statistical Package for the Social Sciences (SPSS) 23.0 software (IBM Corp., Armonk, NY).

Results

A total of 76 scrotal exploration procedures were performed. The involvement of the left side of the scrotum was more common than the right side. Most of the patients who presented were older than five years of age. A majority of the patients presented after 24 hours of the commencement of their symptoms. Of the included participants, 36 patients (47.47%) were found to have an underlying torsion of appendix testes that was appropriately managed. Testicular torsion was observed in 15 patients, out of which eight viable testes were salvaged with a subsequent orchidopexy while seven torsions required orchiectomy owing to their non-viability. Other findings included epididymo-orchitis and infected hydrocele. A total of 19 testes appeared completely normal upon scrotal exploration.

Conclusion

Scrotal exploration should be considered as part of the medical and surgical workup and in the management of acute scrotum, as it divulges the specific underlying testicular pathology. Prompt scrotal exploration can aid in ascertaining the underlying etiology and is, therefore, pivotal in the apt management of the underlying pathology.

## Introduction

Acute scrotum (AS) is a medical and surgical emergency that manifests itself in a multitude of clinical symptoms, including new-onset acute pain, swelling, and tenderness of the scrotum [1]. While the causative etiologies of AS are broad and diverse, possible causes include testicular torsion (TT), epididymo-orchitis (EO), torsion of the appendix testis (TAT), infected hydroceles, and strangulated inguinal hernias [2]. The occurrence and frequency of the aforementioned etiologies vary depending on the age group. In the pediatric population, acute scrotal pain is most often caused by TAT or TT [1]. TT refers to the twisting of the longitudinal axis of the spermatic cord and its contents, compromising the testicular blood flow. TT comprises a pediatric surgical emergency, accounting for nearly 10% to 15% of all the pediatric acute scrotal disease cases [3]. Notably, in the pediatric population, the incidence of TT is 3.6 cases per 100,000 per year, with a majority presenting between the ages of 12 and 16 years [2]. Due to critical and compromised tissue perfusion observed in TT, there is a window of four to eight hours before significant ischemic damage ensues. Therefore, in instances where the prompt management of TT is obtained within the first six hours of the onset of symptoms, testicular salvage rates hover around 90% to 100% [3]. It is, therefore, crucial to promptly diagnose and differentiate TT from other causes of acute scrotal pain. Thus, dilatory management of acute scrotal pain can culminate in adverse disease outcomes, including decreased testicular fertility and testicular non-viability, thereby necessitating an orchiectomy [2].

A comprehensive approach, with an inclusive history, physical examination, and meticulous radiological workup, is vital in aiding the diagnosis of TT. The classic presentation of TT is a sudden onset of unilateral testicular pain [1-3]. In such instances, the associated history is often significant for symptoms such as fever, urinary symptoms, and abdominal pain. Moreover, clinicians should consider querying about precipitating factors, including trauma and a prior history of cryptorchidism. Physical findings may reveal an ipsilateral scrotal skin induration, erythema, or warmth in addition to a high-riding testicle [4]. During palpation, the clinician should also look for any testicular masses, which can also herald the onset of TT. Examiners should also assess for the absence of a cremasteric reflex on the afflicted side [2,5]. A horizontal orientation of the testis, rather than the normal vertical orientation, is one of the most important precipitating factors of TT that can be detected upon physical examination. The horizontal lie of the testis is believed to be caused by an inappropriately high attachment of tunica vaginalis, often referred to as a bell-clapper deformity [5]. The definite treatment for TT draws upon surgical exploration and is often followed by orchiopexy to avoid recurrent torsion. Delayed identification of TT may result in the loss of the affected testicle. Therefore, early recognition of the key signs and symptoms is paramount in avoiding potentially catastrophic disease outcomes. The present study aims to assess the spectrum of testicular pathologies observed during scrotal exploration. The results of the study will aid pediatric surgeons in evaluating and managing this acute condition adeptly.

## Materials and methods

This multicenter, retrospective cross-sectional study was conducted in the departments of pediatric surgery at two hospitals for a duration of three years, from 2017 till 2020. A total of 76 patients aged under 14 who had American Society of Anesthesiologists Score I (ASA-I) were included in the study. All of the included participants had undergone scrotal exploratory procedures necessitated by acute scrotum and were operated under aseptic conditions and total intravenous general anesthesia. Patients with obvious clinical features of TAT, including pain and tenderness with a blue spit sign and positive cremasteric reflex, were excluded from the study. Moreover, patients with explicit clinical signs of EO, including the gradual onset of testicular pain, fever, dysuria, elevated total leukocyte count, and hypervascularity of testis on ultrasound were also excluded. The exclusion criteria ensured that only patients undergoing scrotal exploration for the evaluation of acute scrotum are represented in the study cohort. Additionally, the laterality, age of the patients, and duration of symptoms were assessed. Frequencies of the diverse testicular pathologies, which included TT, TAT, EO, and infected hydrocele, were ascertained upon exploration. The data were collected from patient files and hospital records and were analyzed using the Statistical Package for the Social Science (SPSS) software, version 23 (IBM Corp., Armonk, NY).

## Results

The present study involved 76 male patients who underwent scrotal exploration for their acute scrotum. Left-sided scrotal involvement was more commonly observed than right-sided scrotal involvement. Table 1 delineates the laterality of scrotal involvement within our cohort.

**Table 1:** Breakdown of the patients across laterality

Scrotal involvement	Frequency (n)	Percentage (%)
Left side	42	55.26%
Right side	34	44.74%

A breakdown of the study population with respect to the various age groups is delineated in Table 2.

**Table 2:** A breakdown of study participants with respect to age groups

Age groups	Frequency (n)	Percentage (%)
Neonates (below 1 month of age)	3	3.95%
Infant (1 month to 1 year of age)	8	10.52%
1-5 years	19	25.0%
5-10 years	18	23.68%
10-13 years	28	36.84%

Furthermore, the analysis revealed that a significant proportion of the patients presented with symptoms lasting longer than one day. This is elucidated in Table 3.

**Table 3:** A tabulation of the patients with respect to the duration of their symptoms

Duration of symptoms	Frequency (n)	Percentage (%)
Less than 6 hours	16	21.05%
6-24 hours	20	26.32%
1-2 days	12	15.79%
> 2 days	28	36.84%

**Figure 1:**
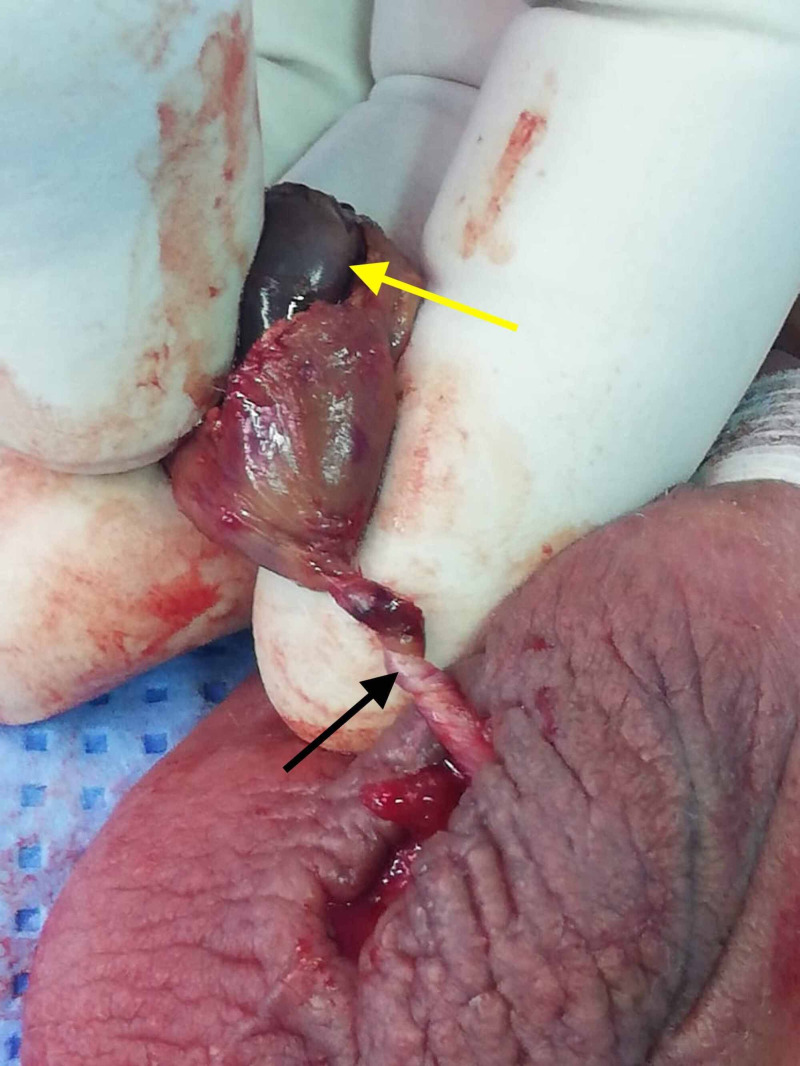
A delineation of the gangrenous testis (yellow arrow) due to testicular torsion with multiple twists of the spermatic cord (black arrow).

Scrotal exploration was performed in all of the patients included in our study and revealed that 15 patients (19.73%) had an underlying TT. Out of these 15 patients, eight patients were noted to have viable testes for which an orchidopexy was subsequently performed. Contrarily, the remaining seven patients had non-viable testes; for these patients, an orchiectomy was performed with fixation of the opposite testicle. The elucidation of a gangrenous testis, as observed in one of these aforesaid patients, is elucidated in Figure 1.

**Figure 2:**
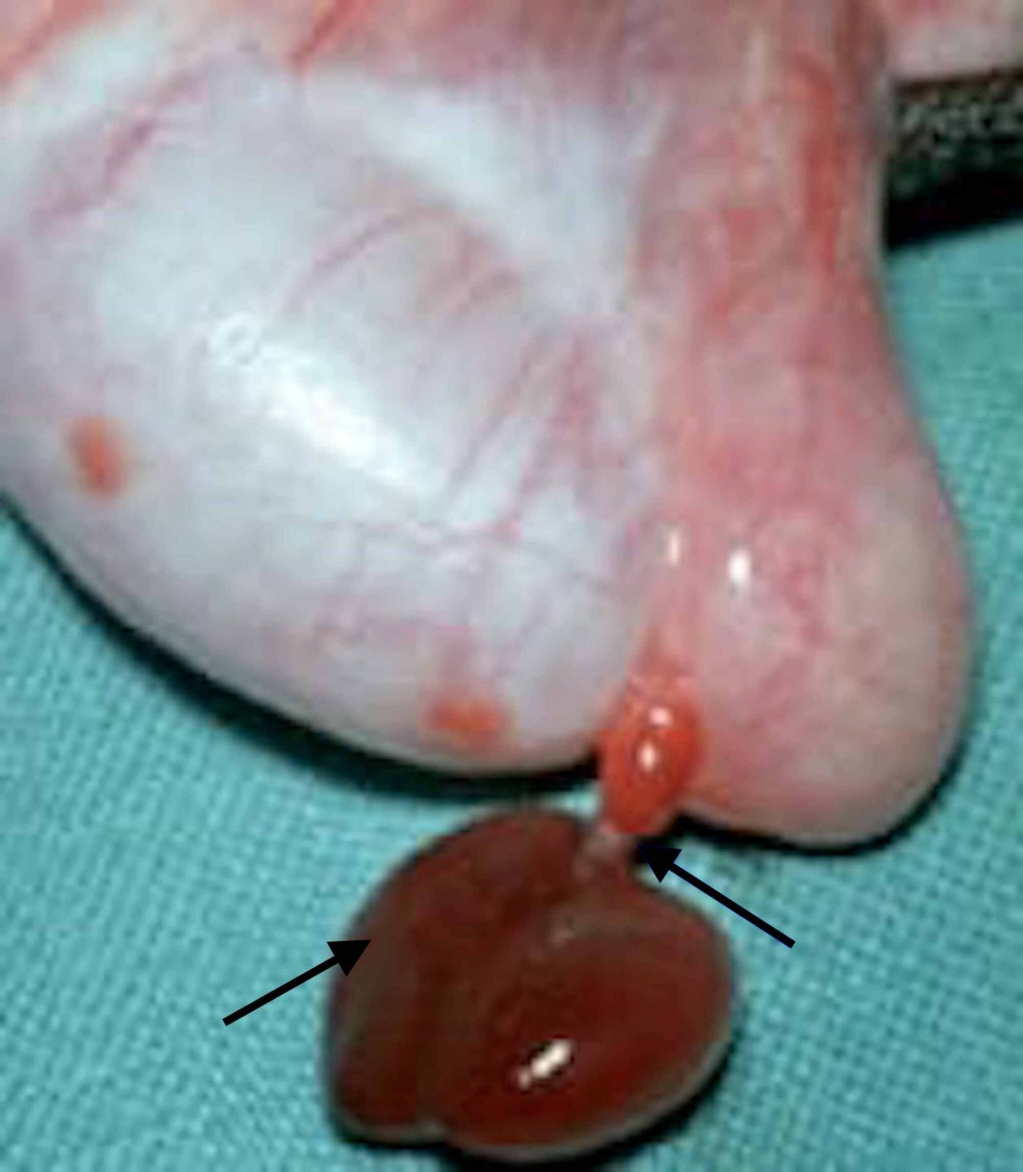
A depiction of torsion of the appendix testis (black arrows)

Furthermore, 36 patients were noted to have an underlying TAT, which was also the most frequently observed pathology in our study. The gross morphology of TAT, as observed in our study, is elucidated in Figure 2. 

Additionally, further analysis divulged that a plethora of the patients had either an underlying TT or TAT. The frequencies and percentages of the various testicular pathologies observed in our study are elucidated in Table 4.

**Table 4:** A tabulation of the diverse spectrum of the various testicular pathologies observed upon scrotal exploration.

Testicular pathology observed	Frequency (n)	Percentage (%)
Testicular torsion	15	19.74%
Torsion of appendix testis	36	47.37%
Epididymo-orchitis	4	5.26%
Infected hydrocele	2	2.63%
Normal testis (no pathology observed)	19	25%

**Figure 3:**
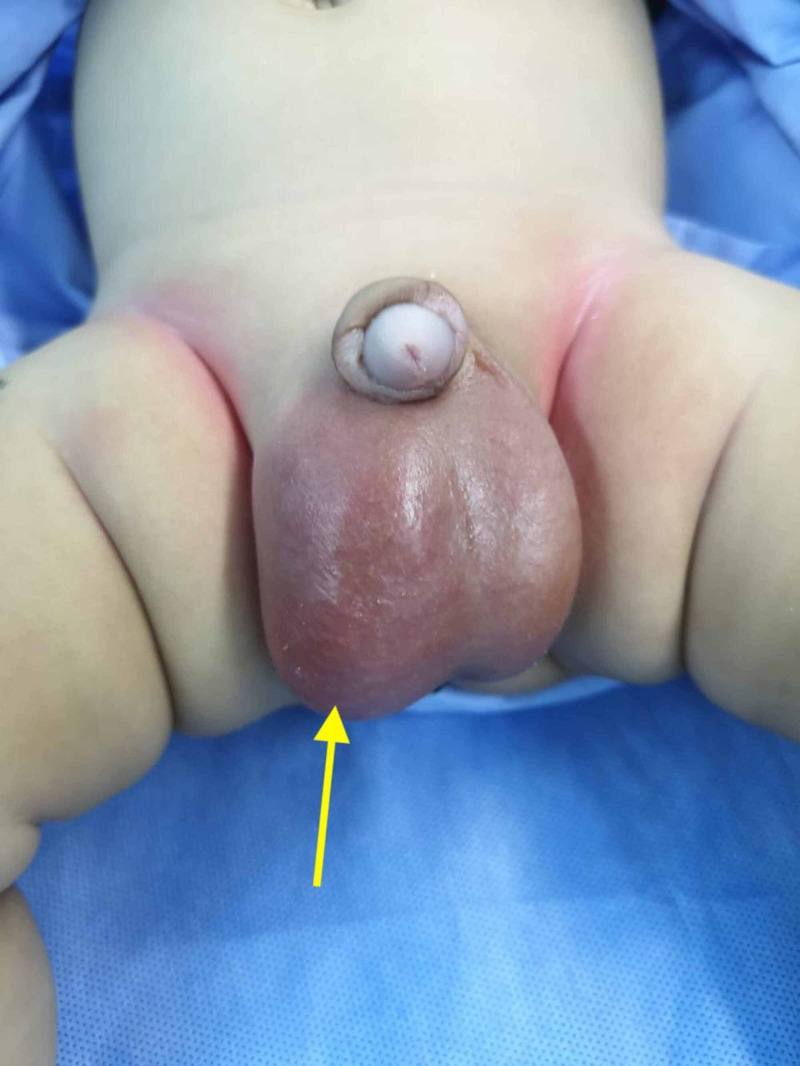
A depiction of bilateral hydrocele with an infected right side (yellow arrow)

While TAT was the most common pathology in the present study, two cases of infected hydrocele were also observed. Figure 3 depicts one of the two cases of bilateral hydrocele in which the right side was predominantly infected.

## Discussion

AS can be caused by a myriad of underlying etiologies, which can be aptly discerned upon scrotal exploration. In the setting of AS, a clinician must take into consideration the possibility of TT and must be able to confidently identify cases of TT. Imperatively, time has been established to be the most significant factor in the management of TT; the success rate of salvaging the testis is 90% to 100% within four to eight hours of the onset of symptoms, 50% at 12 hours, and merely 10% after 24 hours have elapsed [4].

A thorough history and physical examination remain pivotal in aiding the diagnoses of various scrotal pathologies in the setting of AS. TT usually presents as an asymmetrically painful, erythematous, and swollen scrotum [4-5]. On physical examination, a high-riding testis, an abnormally oriented testis, and absent cremasteric reflexes should raise suspicion of TT. The cremasteric reflex is elicited when the medial aspect of the thigh is stroked, leading to the contraction of the cremasteric muscle and thus resulting in the elevation of the testis [6]. In TAT, the necrotic appendix can be observed through the scrotum, giving rise to the blue dot sign. A positive Prehn’s sign, whereby elevation of the scrotum alleviates the pain in the affected testicle, alludes to the diagnosis of epididymitis. Laboratory investigations can be performed to rule out other possible etiologies. Nevertheless, clinicians must not depend exclusively on clinical findings since they are not absolute in yielding a definitive diagnosis, and misdiagnosis of TT can lead to exceedingly adverse disease outcomes such as gangrene of the testis [6-8]. Murphy et al. reported three cases of patients with a normal cremasteric reflex requiring orchidectomy [7]. Additionally, two patients with TT presented entirely pain-free, with painless swelling, but had a tender testis on examination. One of these patients had a short history of swelling for 48 hours with a visible blue spot and an unremarkable ultrasound examination. Thus, these unusual presentations should also be borne in mind when evaluating the underlying etiology.

The color Doppler ultrasound (CDUS), being a very important diagnostic tool, is widely used in the workup of AS, especially in instances where the absence of arterial blood flow in the affected testicle arouses suspicion of TT [9]. However, the presence of arterial flow in TT has been reported previously in the literature [8-9]. It has been reported that CDUS has a sensitivity of 88.9% and specificity of 98.8%, with a 1% rate of false-positive results [9]. Pertinently, Allen et al. documented cases where CDUS findings were inconsistent with findings at surgery [10]. This means that the clinical utility of imaging modalities depends strongly on the operator, and the availability of these imaging modalities in the emergency setting might vary between institutions. Even though a thorough history, complete clinical examination, and radiological investigations are focal in aiding the diagnosis, they are not always reliable in conclusively ruling out TT in the setting of AS. For this reason, there has been great controversy on the choice of immediate scrotal exploration in the setting of AS. Even though TAT is the most common cause of scrotal pain, it is commonly misdiagnosed, with the preoperative diagnosis being correct in only 11% of cases [6-7,11]. To this end, Hastie et al. have advocated for a conservative approach in patients presenting with a palpable, tender nodule and in patients presenting more than 24 hours since the inception of pain, scrotal erythema, and edema [12]. This allows the avoidance of unnecessary surgery and avoids the financial burden of admission. Furthermore, Holland et al. report that 14% of patients managed conservatively developed persistent pain, which prompted surgical excision [13].

Our study also divulges the usefulness of early scrotal exploration in ascertaining the underlying pathologies. Among 76 patients presenting with AS who underwent subsequent scrotal exploration, 47.37% were found to be afflicted with TAT, which was surgically excised. Similarly, findings characteristic of TT were observed in 19.74% of the patients. Upon scrotal exploration, a determination of the viability of the testes was also made; orchidectomy was performed for non-viable testes while orchidopexy was performed for viable testes. Interestingly, merely 2.63% of the patients were noted to be afflicted with an infected hydrocele. These findings are in accordance with findings documented previously in the literature [8,13]. Studies with larger sample sizes can further help in elucidating the outcomes of scrotal exploration in patients presenting with AS and can better inform the debate pertaining to the spectrum of pathological findings.

## Conclusions

AS is a medical and surgical emergency that, if not addressed promptly, can render the testes non-viable. While a myriad of imaging modalities can be used to aid the diagnosis of the underlying pathologies in cases of AS, their efficacy varies widely across institutions. Owing to its ability to delineate the vast spectrum of the underlying testicular pathologies, scrotal exploration should be performed in all cases of AS in the pediatric population. Knowledge of the underlying pathology can help the surgeon in making an informed decision about how to preserve the testes, thereby avoiding complications such as testicular necrosis and thus portending favorable disease outcomes.
